# Significance of Environmental Input Data in Risk Assessment Analyses

**DOI:** 10.3390/jox10020005

**Published:** 2020-12-21

**Authors:** Agnieszka Gruszecka-Kosowska

**Affiliations:** Department of Environmental Protection, Faculty of Geology, Geophysics and Environmental Protection, AGH University of Science and Technology, Al. Mickiewicza 30, 30-059 Krakow, Poland; agnieszka.gruszecka@agh.edu.pl

The environment is becoming more and more polluted. Results of monitoring studies indicate contents of particular xenobiotics in investigated environmental components, i.e., air, water, soil, and plants. If permissible or recommended concentrations are established, comparison of measured contents of particular xenobiotics with threshold values, allows for determination of the environmental component quality or level of pollution and usually, the defined state of contamination refers to adverse environmental and health effects [1,2]. However, these threshold values refer to general information concerning health effects and related risk. The answer for the limitation of this approach is ecological and health risk assessment procedures. Risk assessment procedures give the risk values and risk characterization under the conditions of reliable usage of the recognized environmental components.

Ecological risk assessment (ERA) describes the impact of analyzed xenobiotics on environments being influenced by the investigated sources of pollution [3]. The ERA analysis described by the United States Environmental Protection Agency (US EPA) consists of the following steps [4]: (1) Planning of the ERA assessment includes defining the object at risk, characterizing the investigated source of pollution in the ERA contaminants of emerging concern (CECs) defining pathways and routes of exposure, defining the impact of environmental hazards on investigated objects, and describing ecological effects. (2) The problem formulation phase refers to defining the ecological entity at risk and the predicted negative effects (assessment endpoints). (3) Risk analysis determines which objects are vulnerable and which level does not cause adverse ecological effects using a hazard quotient approach or it determines relevant parameters. (4) Risk characterization interprets the adversity of assessed ecological effects, indicating uncertainties and degrees of confidence. 

Human health risk assessment (HHRA), also developed by the US EPA, describes the probability of adverse health effects in populations that may be exposed to xenobiotics in particular environments under specific exposure scenarios and exposure pathways [5]. The planning process in the HHRA is similar to that in the ERA but it is focused on humans as target receptors. The health risk assessment also consists of four basic steps [6]: (1) Hazard identification and characterization refers to determining types of adverse health effects caused by exposure of investigated xenobiotics. (2) Dose-response assessment describes how probable and severe adverse health effects will be for investigated populations due to exposure of the dose of the investigated xenobiotic. (3) Exposure assessment refers to determining of magnitude, frequency, and duration of exposure of humans to investigated xenobiotics present in the environment during calculation and/or modelling processes. Discussion of uncertainties is also given here. Risk characterization summarizes obtained results in the form of overall conclusions following the TCCR (transparency, clarity, consistency, and reasonableness) principles. The overall risk characterization gives the foundation for risk management and monitoring plans. 

Thus, to perform both ecological risk assessment (ERA) and human health risk assessment (HHRA) procedures, three main sets of data are required to be collected before risk analysis begins (Figure 1). Here, the significance of the environmental dataset will be considered. This set of data is prepared by the environmental scientists and should give reliable data concerning the type of xenobiotic and its content in the investigated environmental component. There are some issues that should be taken into consideration. Firstly, identification of the list of xenobiotics that will be considered in the risk analysis. The analyzed area might be influenced not only by current anthropogenic activities, but also by historical ones that are sometimes difficult to reconstruct. The next issue is that contaminants do not appear singly, but in the real environments there are mixtures of many different xenobiotics at the same time. Furthermore, a particular xenobiotic might not be present in the same form all the time, and its primary form and metabolites might migrate during the investigation, as well as between environmental components. Therefore, knowledge of potential and real routes of migration of xenobiotics in various components of the environment is also important. A further issue is that the total content of xenobiotics in the environment is not a measurable indicator of environmental hazards, or at least, not the only one. Recently, the bioavailability of particular xenobiotics for target organism has become an important issue [7]. With respect to receptor organisms, absorption, metabolism, and the removal of xenobiotics in particular groups of organism is also crucial [8,9]. Detailed knowledge of the above-mentioned issues requires us to perform risk assessment procedures as reliably as possible. 

All of these environmental aspects significantly affect the calculated risk values. This is especially important in cases where results of risk assessment analyses are used to make administrative decisions related to environmental and human health protection. In the case of uncertain risk values the highest risk values should be taken as a binding decision. This is due to the need to respect the conservative risk assessment principle that describes a worst-case scenario. However, such an approach might generate unnecessary financial expense. Only reliable input environmental data will allow for reliable risk analysis results, maximizing health and environmental protection and minimizing financial costs at the same time.

## Figures and Tables

**Figure 1:**
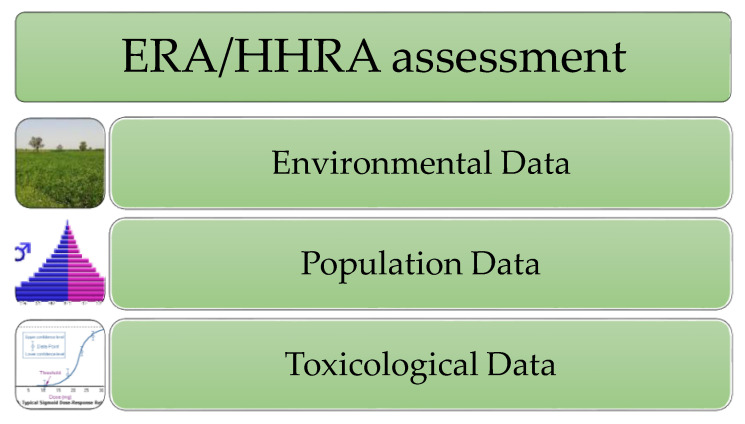
The scheme of the three main input datasets for risk assessment analysis.
